# Exploring the genetic architecture and improving genomic prediction accuracy for mastitis and milk production traits in dairy cattle by mapping variants to hepatic transcriptomic regions responsive to intra-mammary infection

**DOI:** 10.1186/s12711-017-0319-0

**Published:** 2017-05-12

**Authors:** Lingzhao Fang, Goutam Sahana, Peipei Ma, Guosheng Su, Ying Yu, Shengli Zhang, Mogens Sandø Lund, Peter Sørensen

**Affiliations:** 1 Department of Molecular Biology and Genetics, Center for Quantitative Genetics and Genomics, Aarhus University, 8830 Tjele, Denmark; 2 Key Laboratory of Animal Genetics, Breeding and Reproduction, Ministry of Agriculture and National Engineering Laboratory for Animal Breeding, College of Animal Science and Technology, China Agricultural University, Beijing, 100193 China

## Abstract

**Background:**

A better understanding of the genetic architecture of complex traits can contribute to improve genomic prediction. We hypothesized that genomic variants associated with mastitis and milk production traits in dairy cattle are enriched in hepatic transcriptomic regions that are responsive to intra-mammary infection (IMI). Genomic markers [e.g. single nucleotide polymorphisms (SNPs)] from those regions, if included, may improve the predictive ability of a genomic model.

**Results:**

We applied a genomic feature best linear unbiased prediction model (GFBLUP) to implement the above strategy by considering the hepatic transcriptomic regions responsive to IMI as genomic features. GFBLUP, an extension of GBLUP, includes a separate genomic effect of SNPs within a genomic feature, and allows differential weighting of the individual marker relationships in the prediction equation. Since GFBLUP is computationally intensive, we investigated whether a SNP set test could be a computationally fast way to preselect predictive genomic features. The SNP set test assesses the association between a genomic feature and a trait based on single-SNP genome-wide association studies. We applied these two approaches to mastitis and milk production traits (milk, fat and protein yield) in Holstein (HOL, n = 5056) and Jersey (JER, n = 1231) cattle. We observed that a majority of genomic features were enriched in genomic variants that were associated with mastitis and milk production traits. Compared to GBLUP, the accuracy of genomic prediction with GFBLUP was marginally improved (3.2 to 3.9%) in within-breed prediction. The highest increase (164.4%) in prediction accuracy was observed in across-breed prediction. The significance of genomic features based on the SNP set test were correlated with changes in prediction accuracy of GFBLUP (*P* < 0.05).

**Conclusions:**

GFBLUP provides a framework for integrating multiple layers of biological knowledge to provide novel insights into the biological basis of complex traits, and to improve the accuracy of genomic prediction. The SNP set test might be used as a first-step to improve GFBLUP models. Approaches like GFBLUP and SNP set test will become increasingly useful, as the functional annotations of genomes keep accumulating for a range of species and traits.

**Electronic supplementary material:**

The online version of this article (doi:10.1186/s12711-017-0319-0) contains supplementary material, which is available to authorized users.

## Background

In general, genetic variation in complex or quantitative traits is considered to be governed by a large number of loci with small to moderate effects, which are individually undetectable by genome-wide association studies (GWAS) with stringent significance thresholds [1–5]. A better understanding of the genetic architecture that underlies complex traits (e.g. the distribution of causal variants and their effects) could improve the predictive ability of models [4, 6–9]. This would be beneficial for genomic prediction of disease risk in humans and for estimating genetic values in livestock and plant species of agricultural importance [4, 6–9].

The genomic best linear unbiased prediction (GBLUP) assumes that all genomic markers contribute equally to variability of a trait [10] and ignores any prior biological knowledge on genetic architecture of the trait. However, genomic markers that are associated with a complex trait may not be uniformly and randomly distributed over the genome, but rather be clustered in genes that are part of interconnected biological pathways and networks [2, 11, 12]. The genomic regions that are likely to be enriched in variants affecting a trait are defined as genomic features. Based on different biological hypotheses, genomic features can be defined from various sources of biological knowledge, such as genes, gene ontologies, biological pathways, or other types of external evidence. Incorporating this biological information may improve the predictive abilities of models. We extended the GBLUP model to implement this strategy by including a separate random effect for the joint action of single nucleotide polymorphisms (SNPs) within a genomic feature [8], which we call a genomic feature BLUP (GFBLUP) model. As a result, individual SNP relationships can be weighted differentially in GFBLUP according to the variance explained by SNPs within and outside the genomic feature [8]. The GFBLUP model has been applied to three complex traits (i.e. chill coma recovery, starvation resistance and startle response) in the unrelated inbred lines of *Drosophila melanogaster* populations [8]. Compared to GBLUP, the prediction accuracy with GFBLUP was substantially improved when incorporating several gene ontology (GO) categories as genomic features [8]. A possible increase in prediction accuracy with GFBLUP would depend on whether the genomic feature is enriched in causal mutations.

The GFBLUP model is computationally intensive for evaluating many genomic features [8]. Therefore, it is important to develop a computationally fast approach. The SNP set test based on GWAS-derived single-SNP test statistics could be one such approach. It would be of interest to investigate the relationship between the significance of a genomic feature based on the SNP set test and the predictive ability of the GFBLUP model.

To date, there are many genes that are yet neither functionally characterized nor mapped to any biological databases [13–16], in particular in livestock populations. For example, in cattle only ~20% of the genes are annotated in Kyoto Encyclopaedia of Genes and Genomes (KEGG) pathways [17]. However, transcriptomics studies have been conducted on small-scale experimental populations to investigate the dynamic state of the transcriptome in particular tissues, revealing thousands of genomic features (e.g. genes and pathways) that are engaged in the biological processes of complex traits [18–20]. Such transcriptomics studies provide tissue-specific genomic features that are likely to be enriched in genomic variants affecting specific traits.

Mastitis, an inflammatory condition of the mammary gland, is often caused by invading pathogens. It is the most costly disease in the dairy industry due to treatment cost, reduction in milk production and milk quality, and in some cases culling of the affected cows [21]. Gram-negative *Escherichia coli* (*E. coli*) is a common mastitis-causing bacteria [22], and the lipopolysaccharides (LPS) released by *E. coli* induce acute inflammatory responses [23]. Genes with expression levels that are significantly affected during the early stage of infection have also been suggested to be involved in overall metabolism [19, 23–26]. Moreover, it is well established that mastitis is unfavorably correlated with milk production traits [25]. Since liver plays key roles in innate immune response and metabolic regulation [27], we hypothesized that hepatic transcriptomic regions that are responsive to intra-mammary infection (IMI) may be enriched in genomic variants that impact mastitis and milk production traits. Using these regions as genomic features might provide more predictive GFBLUP models compared to the GBLUP model. In addition, since gene expression patterns and molecular interaction networks are consistent across breeds [28], we further hypothesized that the use of transcriptomic data obtained on one breed may contribute to improve genomic prediction in other breeds.

In the current study, mastitis and three milk production traits (i.e. milk, fat and protein yield) from Nordic Holstein (HOL, n = 5056) and Jersey (JER, n = 1231) cattle were analyzed using imputed sequence genotype data (~15 million SNPs) and hepatic transcriptome data from an IMI study. Our main objectives were to apply the GFBLUP model and SNP set test: (1) to investigate the genomic variance explained by transcriptomic regions that are responsive to IMI; (2) to improve the accuracy of within-breed and across-breed genomic prediction using GFBLUP compared to GBLUP; and (3) to investigate the relationship between the predictive ability of GFBLUP and the significance of genomic features based on the SNP set test.

## Methods

### Intra-mammary infection (IMI) study

The IMI experimental design and collection of liver biopsies were reported previously [23, 29]. In brief, eight healthy HOL dairy cows in their first lactation (9 to 12 weeks after calving) were selected for the experiment. The udder quarters of all studied cows were free from mastitis pathogens based on bacteriological examinations. Milk somatic cell count (SCC) for each studied quarter was <100,000. The right front quarter was infected with 200 µg of *E. coli* LPS (0111:B4) (Sigma-Aldrich, Brøndby, Denmark) dissolved in 10 mL of a 0.9% NaCl solution, while the left front quarter was used as a control and challenged with 10 mL of 0.9% NaCl solution only. Clinical signs, data on production traits together with milk and blood parameters associated with LPS infection were recorded throughout the trial and confirmed that mastitis inflammation was induced. Liver biopsies collected 22 h before and 3, 6, 9, 12 and 48 h after LPS infection in three cows were used for RNA extraction. Sampling procedures for liver biopsies were described previously [30]. Finally, 18 RNA-Seq libraries (at each time point with three biological replicates) were sequenced using 100-bp paired-end sequencing in Illumina Hiseq2000 sequencing technology.

### Statistical analysis of RNA-Seq data

Statistical approaches used for analysing RNA-Seq data were described previously [31]. Briefly, sequence reads of each sample were aligned to the bovine reference genome assembly (UMD 3.1), using a sensitive and efficient mapping program based on the seed-and-vote algorithm implemented in the Rsubread package in R/Bioconductor [32] _ENREF_65. The number of reads that were mapped to 24,616 Ensemble genes **(ftp://ftp.ensembl.org/pub/release-86/gtf/bos_taurus) was counted using the function Feature-Counts in the Rsubread package with default settings. The average mapping rate across all samples was approximately 68%. Analysis of differential gene expression was conducted using edgeR [33]. A small number of highly expressed genes in a sample can cause an RNA composition effect, i.e. a substantial proportion of the total library size could be consumed by these highly expressed genes, which results in the remaining genes to be under-sampled [33]. Therefore, the most recommended weighted trimmed means of M-values (TMM) were used to normalize the total count data (i.e. the total library size) between each pair of samples, in order to adjust for RNA composition effect [33]. After normalization of the total library size, a negative binomial generalized linear model (GLM) was applied for each gene, because the count data of genes follow non-normal distributions, which commonly exhibit a quadratic mean–variance relationship [33]. The relevant factors in the experimental design were also adjusted by the GLM, and gene differential expression was determined using a likelihood ratio test [33]. In the GLM model, where the number of reads mapped to gene $g$ in sample $i$ is denoted as $y_{gi}$ and the total number of mapped reads is denoted as $N_{i}$, it is assumed that $y_{gi} \sim \,NB\left( {\mu_{gi} ,\phi_{g} } \right)$, where $\mu_{gi}$ and $\phi_{g}$ are the location and the dispersion parameters of the negative binomial distribution, respectively. To ensure stable inference for each gene, an empirical Bayes method was used to compress gene-wise dispersions towards a common dispersion for all genes [33]. Statistical tests for each analysis were adjusted for multiple-testing using the FDR method as implemented in R (version 3.2.4).

### Defining genomic features

The differentially-expressed genes (DEG) (i.e. the hepatic transcriptome regions responsive to IMI) that were obtained from the above RNA-Seq analyses were used to define genomic features. First, 30 genomic features were defined using six false discovery rate (FDR) cut-off values (i.e. ≤5×10^−2^, 10^−2^, 10^−3^, 10^−6^, 10^−8^, and 10^−10^) in each of the five experimental comparisons (i.e. 3 vs. −22 h, 6 vs. −22 h, 9 vs. −22 h, 12 vs. −22 h and 48 vs. −22 h), respectively. In addition, since the biological functions of up-regulated and down-regulated genes can be quite different, each of these 30 genomic features was further divided into four subsets based on four log_2_(fold-change)s cut-off values (i.e. ≤−2, ≤−1, ≤1, and >2). Therefore, another 115 genomic features were built, because five conditions were without DEG. In total, 145 genomic features were defined. The number of DEG in each genomic feature is summarized in Table S1 (see Additional file 1: Table S1).

#### Phenotypic data

The phenotypes were de-regressed breeding values (DRP) from routine genetic evaluations by the Nordic Cattle Genetic Evaluation (NAV, http://www.nordicebv.info/), and were available for 5056 HOL and 1231 JER cattle. Detailed information of these phenotypes was previously described in [34, 35]. Heritabilities for milk, fat and protein yields and mastitis were equal to 0.39, 0.39, 0.39 and 0.04, respectively in HOL, and very similar in JER [34, 35]. The average reliabilities of the DRP for milk, fat and protein yields and mastitis were equal to 0.95, 0.95, 0.95 and 0.83, respectively in HOL; and 0.92, 0.92, 0.92, and 0.76, respectively in JER.

#### Genotypic data

Imputation from Illumina BovineSNP50 BeadChip (50 K) to Illumina BovineHD BeadChip (high-density, HD) genotypes for these individuals and further to whole-genome sequence variants was described previously [36, 37]. Briefly, genotypes from the 50 K SNP chip for each individual were first imputed to HD genotypes using a multi-breed reference of 3383 animals (1222 HOL, 1326 Nordic Red, and 835 JER). A total of 648,219 SNPs were obtained after imputation to the HD chip. These imputed HD genotypes were then imputed to the whole-genome sequence level using a multi-breed reference population of 1228 individuals from *Run4* of the 1000 Bull Genomes Project [38] and additional whole-genome sequences from Aarhus University including 368 HOL, 86 Nordic Red, and 88 JER individuals [39]. Genotype imputation was done using *Minimac2* [40]. In total, 22,751,039 biallelic variants (SNPs and Indel) were included in the imputed sequence genotypic data. The accuracy of imputation was above 0.85 for the across-breed imputation of 19,498,365 SNPs. Detailed information about imputation accuracy was previously reported in [37]. For each breed, SNPs with a large deviation from Hardy–Weinberg proportions (*P* < 10^−6^) or with minor allele frequency (MAF) <0.01 were further excluded. A total of 15,355,382 and 13,403,916 SNPs remained for the HOL and JER datasets, respectively. The SNP locations were based on the UMD3.1 reference genome (http://www.ensembl.org/Bos_taurus/Info/Index). A SNP was considered to be linked with a genomic feature if its chromosome position was within the open reading frame of DEG in the particular genomic feature.

#### Training and validation populations

For within-breed prediction, each of the datasets (i.e. HOL and JER) was divided into training and validation sets based on birth-year of the animal to access prediction accuracy. The birth-year cut-off was 2006 for HOL and 2004 for JER, and the younger animals were assigned to the validation dataset (Table 1). We chose this validation strategy considering routine animal breeding practice where the young bulls breeding values are predicted using a training population of older animals. For across-breed prediction, the complete HOL population (n = 5056) was used as training data to predict breeding values for all JER bulls (n = 1231). Both GBLUP and GFBLUP models were fitted to compare prediction accuracies.

**Table 1:** Overview of training and validation population sizes for genomic predictions

Breed	Number of training individuals	Number of validation individuals	Total number
Within HOL	4011	1054	5056
Within JER	975	256	1231
Across breeds	5056	1231	6287

### Genomic models

For each genomic feature as defined before, SNPs were partitioned into two sets (i.e. within and outside the genomic feature), followed by the GFBLUP model analysis:

$${\mathbf{y}} = \mathbf{1}\mu + \varvec{ }{\mathbf{g}}_{\text{f}} + {\mathbf{g}}_{{ - {\text{f}}}} + {\mathbf{e}},$$

where ${\mathbf{y}}$ is the vector of phenotypic observations, **1** is a vector of 1s, $\mu$ is the overall mean, ${\mathbf{g}}_{\text{f}}$ is the vector of genomic values captured by the SNPs within a genomic feature, ${\mathbf{g}}_{{ - {\text{f}}}}$ is the vector of genomic values captured by SNPs outside the genomic feature (i.e. the rest of genome), and ${\mathbf{e}}$ is the vector of residuals. Assumptions for all random effects are given by:

$$\left( {\begin{array}{*{20}c} {\begin{array}{*{20}c} {{\mathbf{g}}_{{\text{f}}} } \\ {{\mathbf{g}}_{{ - {\text{f}}}} } \\ \end{array} } \\ {\mathbf{e}} \\ \end{array} } \right)\sim{\mkern 1mu} N\left[ {\left( {\begin{array}{*{20}c} {\begin{array}{*{20}c} 0 \\ 0 \\ \end{array} } \\ 0 \\ \end{array} } \right),\left( {\begin{array}{*{20}c} {{\mathbf{G}}_{{\text{f}}} {\sigma }_{{\text{f}}}^{2} } & 0 & 0 \\ 0 & {{\mathbf{G}}_{{ - {\text{f}}}} {\sigma }_{{ - {\text{f}}}}^{2} } & 0 \\ 0 & 0 & {{\mathbf{D}}{\sigma }_{{\text{e}}}^{2} } \\ \end{array} } \right)} \right],$$

where ${\mathbf{G}}_{\text{f}}$ and ${\mathbf{G}}_{{ - {\text{f}}}}$ are genomic relationship matrices that are built using the SNPs within and outside the genomic feature, respectively, which were calculated using the second method described in [41]. Briefly, let ${\mathbf{M}}$ be the marker matrix that specifies which alleles the individual inherits, and ${\mathbf{P}}$ be the matrix that contains the frequencies of the second allele at locus ($p_{i}$) expressed as a difference from the 0.5 value and multiplied by 2, that is, the column $i$ of ${\mathbf{P}}$ is $2\left( {p_{i} - 0.5} \right)$. Matrix ${\mathbf{Z}}$ was obtained as ${\mathbf{M}} - {\mathbf{P}}$, which allows mean values of the allele effects to be equal to 0. Then, ${\mathbf{G}} = {\mathbf{ZTZ}}^{{\prime }}$, where ${\mathbf{T}}$ is a diagonal matrix with $T_{ii} = \frac{1}{{m\left[ {2p_{i} \left( {1 - p_{i} } \right)} \right]}}$. ${\mathbf{D}}$ is a diagonal matrix with diagonal elements equal to $\frac{{1 - r^{2} }}{{r^{2} }}$, where $r^{2}$ is the reliability of DRP, $\upsigma_{\text{f}}^{2}$,$\upsigma_{{ - {\text{f}}}}^{2}$ and $\upsigma_{\text{e}}^{2}$ are the variance components accounted for by the SNPs within and outside the genomic feature, and by the residuals, respectively.

The standard GBLUP model includes only one random genomic effect:

$${\mathbf{y}} = \mathbf{1}\mu + {\mathbf{g}} + {\mathbf{e}},$$

with the same notation as above except for ${\mathbf{g}}$, which is the vector of genomic values captured by all genomic SNPs. The random genomic values and the residuals were assumed to be independently distributed: ${\mathbf{g}} \sim \,N\left( {{\mathbf{0}},{\mathbf{G}}\upsigma_{\text{g}}^{2} } \right)$ and ${\mathbf{e}} \sim \,N\left( {{\mathbf{0}},{\mathbf{D}}\upsigma_{\text{e}}^{2} } \right)$.

#### Estimation of genomic parameters

The variance components, $\upsigma_{\text{f}}^{2}$, $\upsigma_{{ - {\text{f}}}}^{2}$, $\upsigma_{\text{g}}^{2}$ and $\upsigma_{\text{e}}^{2}$, were estimated using an average information restricted maximum-likelihood (AI-REML) procedure [42] implemented in DMU [43]. The proportion of genomic variance explained by a genomic feature in the GFBLUP model: $H_{\text{f}}^{2} = \frac{{\upsigma_{\text{f}}^{2} }}{{\upsigma_{\text{f}}^{2} +\upsigma_{{ - {\text{f}}}}^{2} }}$. The proportion of phenotypic variance explained by all SNPs: $h_{\text{GFBLUP}}^{2} = \frac{{\upsigma_{\text{f}}^{2} +\upsigma_{{ - {\text{f}}}}^{2} }}{{\upsigma_{\text{f}}^{2} +\upsigma_{{ - {\text{f}}}}^{2} +\upsigma_{\text{e}}^{2} }}$ for GFBLUP, and $h_{\text{GBLUP}}^{2} = \frac{{\upsigma_{\text{g}}^{2} }}{{\upsigma_{\text{g}}^{2} +\upsigma_{\text{e}}^{2} }}$ for GBLUP.

#### Validation of genomic prediction

Genomic breeding values (GEBV) were predicted using both GFBLUP and GBLUP models. In the GFBLUP and GBLUP models, GEBV is ${\hat{\mathbf{g}}}_{total} = {\hat{\mathbf{g}}}_{\text{f}} + {\hat{\mathbf{g}}}_{{ - {\text{f}}}}$ and ${\hat{\mathbf{g}}}_{total} = {\hat{\mathbf{g}}}$, respectively. Accuracy of predicted genomic breeding values (*r*) is calculated as the correlation between GEBV and DRP in the validation population. The bias of the genomic predictions with both GFBLUP and GBLUP was evaluated by the regression of DRP on the GEBV, i.e. ${\text{bias}} = {\text{cov}}\left( {{\text{DRP}}, {\text{GEBV}}} \right)/\upsigma_{\text{GEBV}}^{2}$.

### Single-marker GWAS

Single-marker GWAS analyses for four traits were only conducted in the HOL training population, followed by SNP set test analyses for testing the associations between genomic features and traits. Single-marker GWAS was performed using a two-step variance component-based method, to account for population stratification, as implemented in EMMAX [44]. In the first step, the polygenic and residual variances were estimated using the following model:

$${\mathbf{y}} = \mathbf{1}\mu + {\mathbf{a}} + {\mathbf{e}},$$

where ${\mathbf{y}}$ is a vector of phenotypes; $\mathbf{1}$ is a vector of 1s; $\mu$ is the overall mean; ${\mathbf{a}}$ is a vector of breeding values, where ${\mathbf{a}} \sim \,N\left( {0,{\mathbf{G}}\upsigma_{\text{a}}^{2} } \right)$, and ${\mathbf{G}}$ is the genome relationship matrix estimated using EMMAX based on HD SNP genotypes, but excluding the SNPs on the chromosome that harbours the SNP the effect of which is being estimated; and ${\mathbf{e}}$ is the vector of residuals, where ${\mathbf{e}} \sim \,N\left( {0,{\mathbf{I}}\upsigma_{\text{e}}^{2} } \right)$ and ${\mathbf{I}}$ is an identity matrix. In the second step, the individual effects of SNPs were obtained using a linear regression model:

$${\mathbf{y}} = \mathbf{1}\mu + {\mathbf{xb}} + {\varvec{\upeta}},$$

where ${\mathbf{y}}$, $\mathbf{1}$ and $\mu$ are as defined above; ${\mathbf{x}}$ is a vector of imputed genotype dosages (ranging from 0 to 2), ${\mathbf{b}}$ is the vector of allele substitution effects ($b$), and ${\varvec{\upeta}}$ is a vector of random residual deviates with (co)variance structure ${\mathbf{G}}\upsigma_{\text{a}}^{2} + {\mathbf{I}}\upsigma_{\text{e}}^{2}$.

### SNP set test

#### Summary statistic for a genomic feature

The summary statistic of a genomic feature was calculated as the sum of the test statistics (i.e. $t^{2}$) of all SNPs within DEG (i.e. open reading frame) that belonged to the genomic feature:

$$T_{sum} = \mathop \sum \limits_{i = 1}^{{m_{f} }} t_{m}^{2} ,$$

where $m_{f}$ is the number of SNPs located in a genomic feature, and $t_{m}^{2}$ is the square of the $t$-statistics for each SNP in the genomic feature. The $t$-statistics was calculated as the estimate of the SNP effect (i.e. $b$) from single-marker GWAS divided by its standard error. This summary statistic is more powerful compared to count-based summary statistics, particularly in situations where genomic features harbor many SNPs each having a small to moderate effect [9, 45].

#### Testing for association between a genomic feature and a trait

Under the null hypothesis, all SNPs in a genome feature have the same joint effect as those in the randomly selected genomic features. To ensure a null hypothesis is competitive to the alternative hypothesis, the random genomic features must contain the same number of SNPs as the genomic feature being analysed, and the linkage disequilibrium (LD) structure among SNPs should be retained. An empirical distribution of the summary statistics of a genomic feature was therefore obtained by using the following cyclical permutation procedure as described previously [9, 46]. Briefly, the test statistics of SNPs (i.e. $t^{2}$) were first ordered based on the chromosome position of the SNPs. A test statistic was randomly selected from this vector. All test statistics were then shifted to new positions, where the selected SNP became the first one, and the other SNPs shifted to new positions, but retained their original order. This uncouples any associations between SNPs and the genomic feature, while retaining the LD structure among SNPs. A new summary statistic was then calculated according to the original position of the genomic feature. The permutation was repeated 1000 times for each genomic feature, and an empirical *P* value was then calculated based on one-tailed tests of the proportion of randomly sampled summary statistics that were larger than that observed.

### Biological function enrichment analysis

In order to investigate the biological function of a genomic feature, functional enrichment analysis of DEG in the particular genomic feature was conducted using a web-based tool, KOBAS2.0 (http://kobas.cbi.pku.edu.cn/home.do) [47], where a hypergeometric gene set enrichment test, based on a gene ontology (GO) database, was applied. The FDR method [48] was used for adjusting multiple tests.

## Results

The results for RNA-Seq analyses at different time-point comparisons (i.e. 3 vs. −22 h, 6 vs. −22 h, 9 vs. −22 h, 12 vs. −22 h and 48 vs. −22 h) are summarized in Table S2 (see Additional file 2: Table S2). The −log_10_(*P*) values of imputed sequence-level SNPs from single-marker GWAS for mastitis and milk production traits on the HOL training population are shown in the Manhattan plots of Figure S1 (see Additional file 3: Figure S1). The GFBLUP and GBLUP models were compared for all four traits in within-breed (i.e. HOL and JER) genomic prediction, followed by across-breed prediction (i.e. HOL as the training population and JER as the validation population). The degree of enrichment (i.e. −log_10_(*P* values)) of genomic features based on the SNP set test in the HOL training population was compared with the changes in prediction accuracy of GFBLUP within- and across-breed predictions, respectively.

### GBLUP, GFBLUP and SNP set test analyses for Holstein population

#### Genomic parameters

**Fig. 1:**
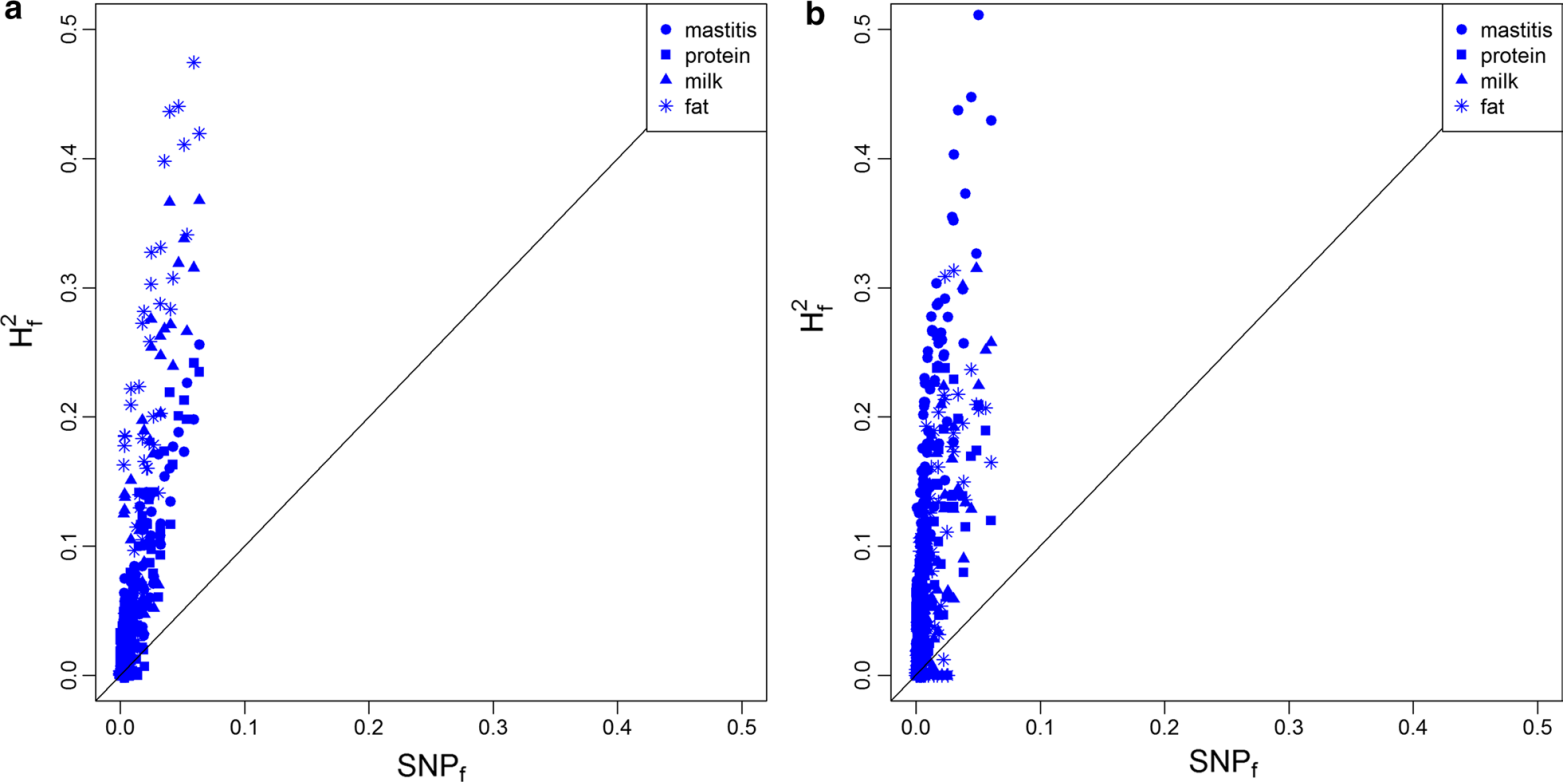
Proportion of genomic variance explained by the genomic features. *Each point* represents one of the 145 genomic features. **a** is for Holstein; **b** is for Jersey; the *x axis* represents the proportion of SNPs over the whole genome that are located in genomic features (i.e. SNP_f_); the *y axis* represents the proportion of genomic variance explained by the genomic features (i.e. ${\text{H}}_{\text{f}}^{2}$)

As shown in Fig. 1a, 128, 106, 99, and 90 of the 145 genomic features explained larger proportions of the total genomic variance ($H_{f}^{2}$) compared to their SNP-proportion over the whole genome for mastitis, protein, milk and fat yield, respectively. Detailed information is summarized in Tables S3, S4, S5 and S6 (see Additional file 4: Tables S3, S4, S5 and S6). These results demonstrated that the genomic variance of the traits studied is not uniformly distributed along the genome, but appears to be enriched in a subset of hepatic transcriptomic regions that are responsive to IMI. Therefore, the assumption of the GBLUP approach that a priori all markers contribute equally to trait variability does not hold good.

#### Prediction accuracy

**Fig. 2:**
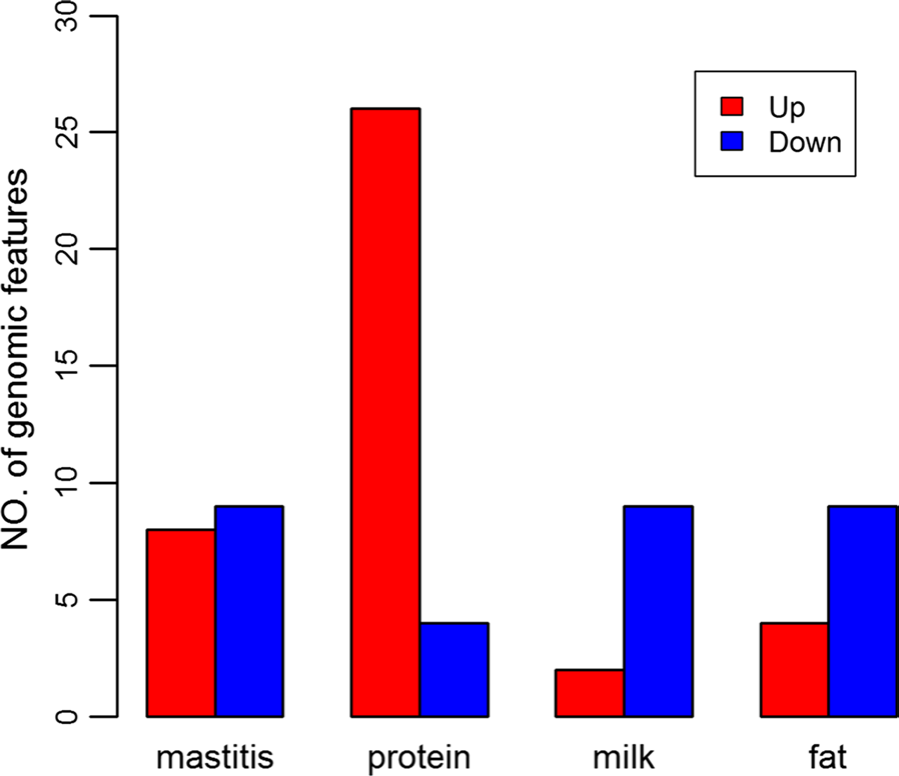
Number of up- (down-) regulated genomic features that result in higher prediction accuracy (Δ*r* > 0.01) with GFBLUP in Holstein population. *Up* represents up-regulated genomic features; *down* represents down-regulated genomic features

Prediction accuracy of GBLUP was equal to 0.504 (bias = 0.864) for mastitis, 0.602 (bias = 0.775) for protein yield, 0.635 (bias = 0.862) for milk yield, and 0.607 (bias = 0.808) for fat yield. Compared to the GBLUP model, 27, 44, 17 and 13 of the 145 genomic features resulted in higher prediction accuracies with GFBLUP (Δ*r* ≥ 0.01) for mastitis, protein, milk and fat yield, respectively (see Additional file 4: Tables S3, S4, S5 and S6). Among these, we found 8 (9) up- (down-) regulated genomic features for mastitis, 26 (4) for protein yield, 2 (9) for milk yield, and 4 (9) for fat yield (Fig. 2). These results indicate that down-regulated genes could be more often associated with milk and fat yield than up-regulated genes during IMI. The regression coefficient of DRP on GEBV (bias) for all GFBLUP analyses ranged from 0.862 to 0.873 for mastitis, from 0.772 to 0.783 for protein yield, from 0.857 to 0.866 for milk yield, and from 0.778 to 0.821 for fat yield (see Additional file 4: Tables S3, S4, S5 and S6). The absolute value of (1-bias) tended to be negatively correlated with the change in genomic prediction accuracy with GFBLUP across four traits (see Additional file 5: Figure S2), which indicates that more predictive genomic features lead to less biased predictions. The top five predictive genomic features for each of the four traits are presented in Table 2. The average increase in prediction accuracy with the best-performing genomic feature across the four traits was 0.018, which corresponds to an increase of 3.2% relative to GBLUP.

**Table 2:** Top five predictive genomic features for mastitis, protein, milk and fat yield in Holstein cattle

Trait	Time (h)^a^	${\text{FDR}}_{\exp }^{\text{b}}$	Log_2_(FC)^c^	${\text{P}}_{\text{set - test}}^{\text{d}}$	SNP_f_ (%)^e^	${\text{H}}_{\text{f}}^{2}$ (%)^f^	$r_{\text{GFBLUP}}^{\text{g}}$	*bias* ^h^	Δ*r* ^i^
Mastitis	9	5 × 10^−2^	NA^j^	0.013	6.36	25.60	0.520	0.872	0.016
	9	5 × 10^−2^	>1	0.027	2.32	13.71	0.519	0.872	0.015
	6	5 × 10^−2^	NA	0.040	5.92	19.81	0.519	0.873	0.015
	6	10^−2^	NA	0.043	4.68	18.83	0.518	0.871	0.014
	6	10^−3^	NA	0.034	3.54	15.39	0.518	0.871	0.014
Protein	48	10^−6^	>2	0.021	<0.01	1.85	0.622	0.783	0.020
	48	10^−8^	>2	0.029	<0.01	1.75	0.621	0.782	0.019
	48	10^−2^	>2	0.023	0.02	3.28	0.621	0.779	0.019
	48	10^−8^	>1	0.027	<.01	1.71	0.621	0.782	0.019
	48	10^−10^	>2	0.026	<0.01	1.37	0.620	0.782	0.018
Milk	6	10^−2^	NA	0.026	4.68	31.90	0.651	0.863	0.016
	6	10^−3^	NA	0.027	3.54	26.82	0.651	0.865	0.016
	6	10^−3^	<−1	0.024	1.76	19.74	0.650	0.862	0.015
	6	10^−6^	<−2	0.022	0.28	12.49	0.649	0.866	0.014
	6	10^−2^	<−1	0.030	2.49	25.39	0.649	0.859	0.014
Fat	6	10^−6^	<−2	0.027	0.28	16.28	0.629	0.804	0.022
	6	10^−3^	<−2	0.028	0.33	17.76	0.626	0.800	0.019
	6	10^−2^	<−2	0.032	0.36	18.57	0.625	0.798	0.018
	6	5 × 10^−2^	<−2	0.032	0.37	18.51	0.625	0.799	0.018
	9	10^−6^	>1	0.055	0.84	20.94	0.621	0.815	0.014

^a^Time points post intra-mammary infection with *E. coli* LPS

^b^FDR values used to define genomic features from RNA-Seq analysis

^c^Log_2_(fold-change) values used to define up- (down-) regulated genomic features from RNA-Seq analysis

^d^P values from SNP set test on HOL training population

^e^Proportion of SNPs in genomic features over the whole genome

^f^Proportion of the total genomic variance explained by genomic features

^g^Prediction accuracy with GFBLUP

^h^The regression coefficient of de-regressed proofs (DRP) on predicted genomic breeding values (GEBV)

^i^The change of prediction accuracy with GFBLUP relative to GBLUP

^j^The genomic feature defined without log_2_(fold-change)

#### Comparisons between degrees of enrichment based on the SNP set test and changes in prediction accuracy of GFBLUP

**Fig. 3:**
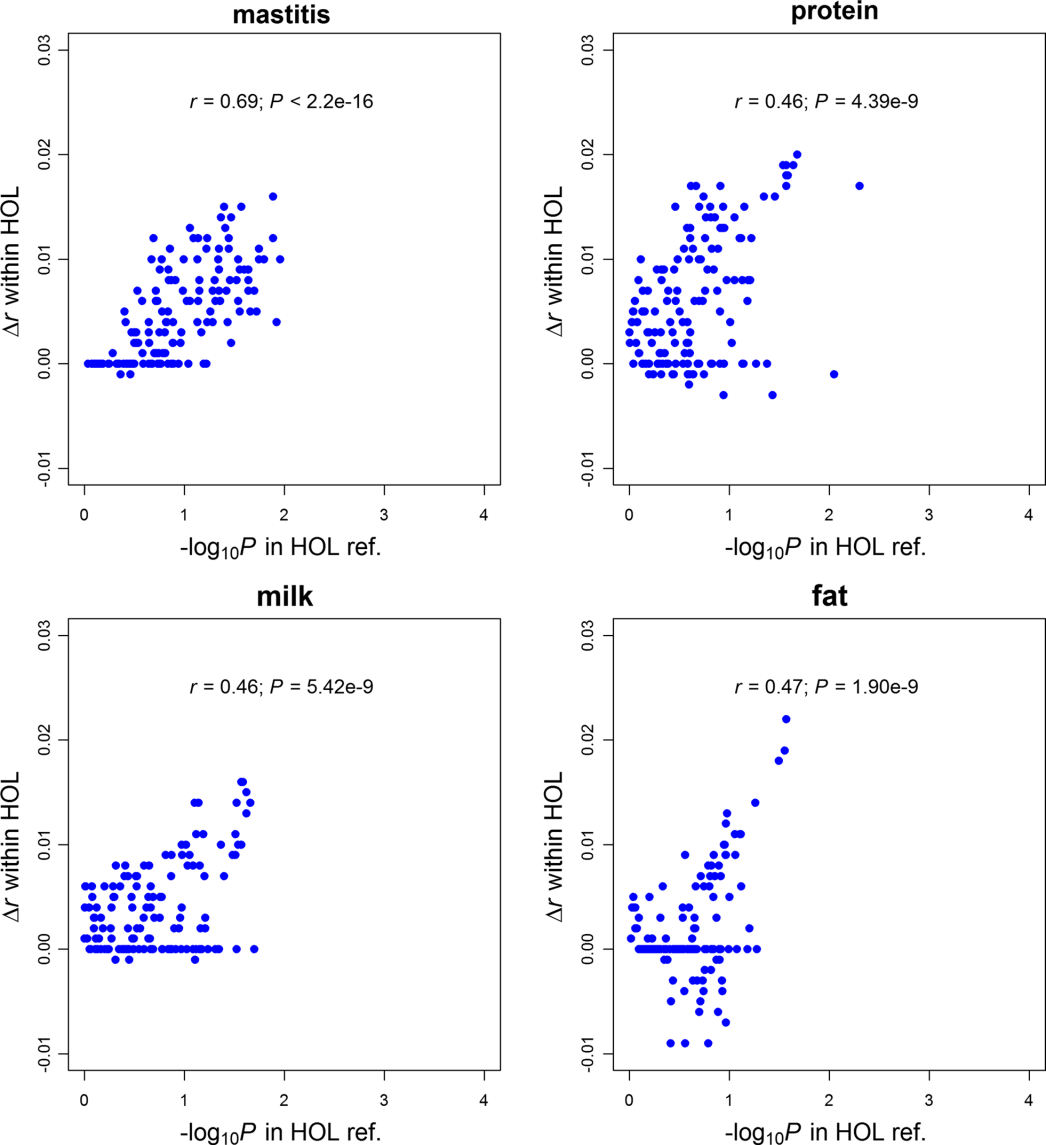
Comparisons between degree of enrichment from the SNP set test in the Holstein (HOL) training (reference) population and changes in prediction accuracy with GFBLUP in the HOL validation population. *Each point* represents one of the 145 genomic features

The results of SNP set tests for all 145 genomic features across four traits in the HOL training population are summarized in Tables S3, S4, S5 and S6 (see Additional file 4: Tables S3, S4, S5 and S6). The changes in prediction accuracy of GFBLUP (Δ*r*) were significantly (*P* < 0.05) positively correlated with –log_10_(*P*) of genomic features based on the SNP set test across all four traits (Fig. 3). Correlations of 0.69 (*P* < 2.2 × 10^−16^), 0.46 (*P* = 4.4 × 10^−9^), 0.46 (*P* = 4.4 × 10^−9^) and 0.44 (*P* = 3.6 × 10^−8^) were found between changes in accuracy and −log_10_(*P* value) for mastitis, protein yield, milk yield, and fat yield, respectively. These results demonstrated that the SNP set test could be used as a computationally simple way to develop more predictive GFBLUP models.

### GBLUP and GFBLUP analyses for the Jersey population

#### Genomic parameters

As in the analyses for the HOL population (Fig. 1b), we observed that 125, 115, 99, and 83 of the 145 genomic features for the JER population explained a larger proportion of the total genomic variance relative to their SNP-proportion over the whole genome for mastitis, protein yield, milk yield, and fat yield, respectively. Detailed information is in Tables S7, S8, S9 and S10 (see Additional file 6: Tables S7, S8, S9 and S10). It should be noted that all genomic features were defined based on gene expression data that were obtained in HOL cattle. These results imply that a subset of hepatic transcriptomic regions responsive to IMI found for HOL were also enriched in genomic variants for mastitis, protein, milk and fat yield in JER.

#### Prediction accuracy

Prediction accuracy of the GBLUP model was equal to 0.549 (bias = 0.916) for mastitis, 0.530 (bias = 0.760) for protein yield, 0.597 (bias = 0.796) for milk yield, and 0.433 (bias = 0.669) for fat yield. Compared to the GBLUP model, 21, 14 and 2 genomic features resulted in higher prediction accuracy (Δ*r* ≥ 0.01) with GFBLUP for mastitis, protein, and milk yield, respectively (see Additional file 6: Tables S7, S8, S9 and S10), among which 7, 13 and 0 were in common with those found for HOL, respectively. No genomic features resulted in an increase >0.005 in prediction accuracy for fat yield in JER. The regression coefficient of DRP on GEBV (i.e. bias) for all the GFBLUP analyses ranged from 0.891 to 0.930 for mastitis, from 0.727 to 0.807 for protein yield, from 0.760 to 0.809 for milk yield, and from 0.599 to 0.677 for fat yield. As observed in HOL, the absolute value of (1-bias) was negatively correlated with the change in prediction accuracy for all four traits in JER (see Additional file 7: Figure S3). The top five predictive genomic features for each of the four traits are summarized in Table 3. The average increase in prediction accuracy (Δ*r*) with the best-performing genomic feature across the four traits was 0.020, which corresponds to a 3.9% increase compared to GBLUP. These results indicate that the use of gene expression data obtained from one breed may improve marginally the genomic prediction accuracy in other breeds. It should be noted that, for JER, the increase in prediction accuracy with GFBLUP for milk and fat yield was very small (Table 3).

**Table 3:** Top five predictive genomic features for mastitis, protein, milk and fat yield in Jersey cattle

Trait	Time (h)^a^	${\text{FDR}}_{\exp }^{\text{b}}$	Log_2_(FC)^c^	SNP_f_ (%)^d^	${\text{H}}_{\text{f}}^{2}$ (%)^e^	$r_{\text{GFBLUP}}^{\text{f}}$	*bias* ^g^	Δ*r* ^h^
Mastitis	9	10^−10^	>1	0.46	15.79	0.567	0.927	0.018
	12	10^−2^	NA^i^	3.98	37.31	0.566	0.930	0.017
	9	10^−10^	NA	1.31	26.64	0.564	0.921	0.015
	12	10^−10^	<−1	0.71	16.15	0.564	0.925	0.015
	6	10^−3^	<−1	1.67	28.69	0.563	0.923	0.014
Protein	48	10^−2^	>2	0.02	6.42	0.576	0.807	0.046
	48	10^−6^	>2	<0.01	4.59	0.571	0.797	0.041
	48	10^−10^	>2	<0.01	4.11	0.569	0.787	0.039
	48	10^−8^	>2	<0.01	4.28	0.569	0.796	0.039
	48	5 × 10^−2^	>2	0.03	6.74	0.568	0.804	0.038
Milk	48	0.01	>2	0.02	2.19	0.608	0.805	0.011
	9	10^−2^	<−1	3.02	12.85	0.607	0.801	0.010
	12	10^−8^	<−1	0.88	10.39	0.606	0.809	0.009
	48	5 × 10^−2^	>2	0.03	1.38	0.605	0.805	0.008
	9	10^−3^	<−1	2.31	13.94	0.604	0.800	0.007
Fat	48	5 × 10^−2^	>1	0.30	4.04 × 10^−7^	0.438	0.672	0.005
	6	5 × 10^−2^	>1	2.57	2.00 × 10^−7^	0.437	0.672	0.004
	48	5 × 10^−2^	NA	0.35	2.24 × 10^−6^	0.437	0.672	0.004
	9	10^−6^	>2	0.32	5.93 × 10^−7^	0.437	0.672	0.004
	9	10^−8^	>2	0.28	5.68 × 10^−7^	0.437	0.672	0.004

^a^Time points post intra-mammary infection with *E. coli* LPS

^b^FDR values used to define genomic features from RNA-Seq analysis

^c^Log_2_(fold-change) values used to define up- (down-) regulated genomic features from RNA-Seq analysis

^d^Proportion of SNPs in genomic features over the whole genome

^e^Proportion of the total genomic variance explained by genomic features

^f^Prediction accuracy with GFBLUP

^g^The regression coefficient of de-regressed proofs (DRP) on predicted genomic breeding values (GEBV)

^h^The change of prediction accuracy with GFBLUP relative to GBLUP

^i^The genomic feature defined without log_2_(fold-change)

#### Comparisons between degree of enrichment from the SNP set test and changes in prediction accuracy of GFBLUP

**Fig. 4:**
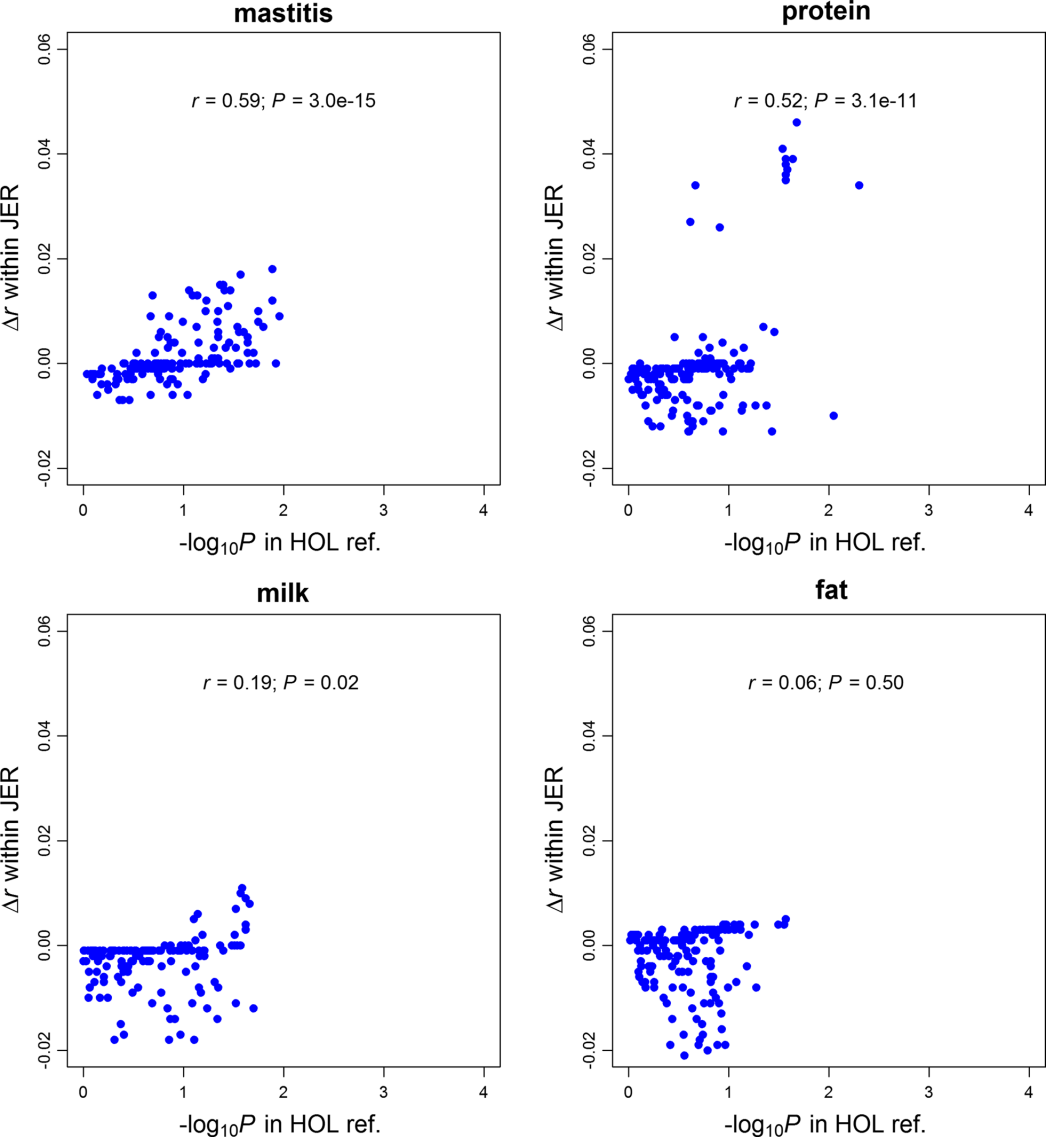
Comparisons between degree of enrichment from the SNP set test in the Holstein (HOL) training (reference) population and changes in prediction accuracy with GFBLUP in the Jersey (JER) validation population. *Each point* represents one of the 145 genomic features

The changes in prediction accuracy with GFBLUP on the JER validation population were also significantly positively correlated with −log_10_(*P*) based on the SNP set test on the HOL training population for mastitis and protein yield (Fig. 4). Correlations of 0.59 (*P* = 3.0 × 10^−15^), 0.52 (*P* = 3.1 × 10^−11^), 0.19 (*P* = 0.02) and 0.06 (*P* = 0.5) were found between changes in accuracy and −log_10_(*P*) for mastitis, protein yield, milk yield, and fat yield, respectively.

### GBLUP and GFBLUP for across-breed genomic prediction

**Fig. 5:**
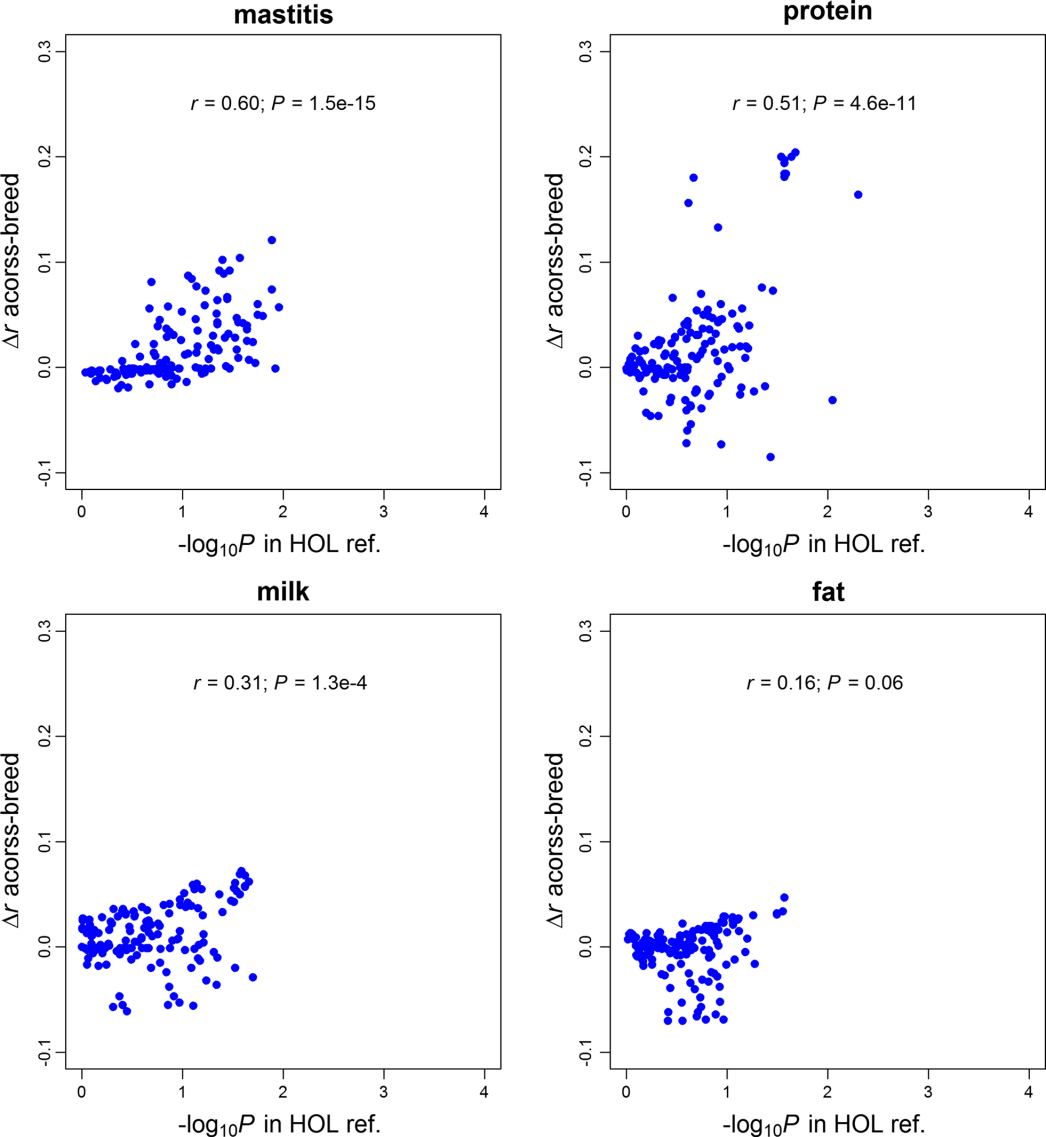
Comparisons between degree of enrichment from the SNP set test in the Holstein (HOL) training (reference) population and changes in prediction accuracy with GFBLUP in the across-breed prediction. *Each point* represents one of the 145 genomic features

When the complete HOL population was considered as training population to predict the genomic values of individuals in the JER population, prediction accuracy of GBLUP was very low, i.e. prediction accuracies were equal to −0.058 (bias = −0.343) for mastitis, 0.098 (bias = 0.622) for protein yield, 0.160 (bias = 0.762) for milk yield, and 0.070 (bias = 0.482) for fat yield. Compared to the GBLUP model, 60, 68, 71 and 44 of the 145 genomic features resulted in higher prediction accuracy with GFBLUP (Δ*r* ≥ 0.01) for mastitis, protein, milk and fat yield, respectively (see Additional file 8: Tables S11, S12, S13 and S14). The regression coefficient (i.e. bias) of DRP on GEBV for all GFBLUP analyses ranged from −0.463 to 0.277 for mastitis, from 0.151 to 1.265 for protein yield, from 0.413 to 0.826 for milk yield, and from 0.002 to 0.577 for fat yield. It should be noted that more predictive genomic features lead to less biased predictions across the four traits (see Additional file 9: Figure S4). In addition, for mastitis, protein and milk yield, the changes in accuracy with GFBLUP in across-breed prediction were significantly correlated with the −log_10_(*P*) of SNP set test in the HOL training population (Fig. 5). The top five predictive genomic features for each of the four traits are summarized in Table 4. The absolute average increase in prediction accuracy (Δ*r*) with the best-performing genomic feature across four traits was 0.111, which corresponds to a 164.4% increase relative to GBLUP. Compared to within-breed prediction, the relative improvement in genomic prediction accuracy seems to be clearer in across-breed prediction.

**Table 4:** Top five predictive genomic features for mastitis, protein, milk and fat yield in across-breed prediction

Trait	Time (h)^a^	${\text{FDR}}_{\exp }^{\text{b}}$	Log_2_(FC)^c^	SNP_f_ (%)^d^	${\text{H}}_{\text{f}}^{2}$ (%)^e^	$r_{\text{GFBLUP}}^{\text{f}}$	*bias* ^g^	Δ*r* ^h^
Mastitis	6	10^−3^	<−1	1.94	9.98	0.063	0.277	0.121
	6	5 × 10^−2^	<−1	3.53	14.03	0.046	0.178	0.104
	6	10^−2^	<−1	2.72	12.68	0.044	0.171	0.102
	9	5 × 10^−2^	NA^i^	6.99	25.98	0.034	0.115	0.092
	12	5 × 10^−2^	>1	2.34	12.84	0.034	0.112	0.092
Protein	48	10^−6^	>2	0.01	2.24	0.302	1.250	0.204
	48	10^−8^	NA	0.01	2.04	0.298	1.264	0.200
	48	10^−8^	>2	<0.01	2.09	0.295	1.265	0.197
	48	10^−3^	>2	0.01	2.66	0.292	1.245	0.194
	48	10^−10^	NA	<0.01	1.60	0.282	1.172	0.184
Milk	9	10^−3^	<−1	2.69	24.65	0.232	0.798	0.072
	9	10^−6^	NA	2.60	14.41	0.229	0.805	0.069
	9	10^−6^	<−1	1.67	8.20	0.228	0.808	0.068
	48	10^−6^	>2	0.01	0.25	0.222	0.826	0.062
	12	10^−8^	<−1	1.02	3.95	0.221	0.802	0.061
Fat	6	10^−3^	>1	1.98	19.66	0.117	0.577	0.047
	9	10^−6^	NA	2.61	24.48	0.104	0.477	0.034
	6	10^−6^	<−1	0.95	20.29	0.102	0.446	0.032
	3	5 × 10^−2^	>2	0.11	0.85	0.101	0.567	0.031
	3	10^−2^	>2	0.11	0.72	0.100	0.560	0.030

^a^Time points post intra-mammary infection with *E. coli* LPS

^b^FDR values used to define genomic features from RNA-Seq analysis

^c^Log_2_(fold-change) values used to define up- (down-) regulated genomic features from RNA-Seq analysis

^d^Proportion of SNPs in genomic features over the whole genome

^e^Proportion of the total genomic variance explained by genomic features

^f^Prediction accuracy with GFBLUP

^g^The regression coefficient of de-regressed proofs (DRP) on predicted genomic breeding values (GEBV)

^h^The change of prediction accuracy with GFBLUP relative to GBLUP

^i^The genomic feature defined without log_2_(fold-change)

### Discovery of gene sets associated with protein yield

**Fig. 6:**
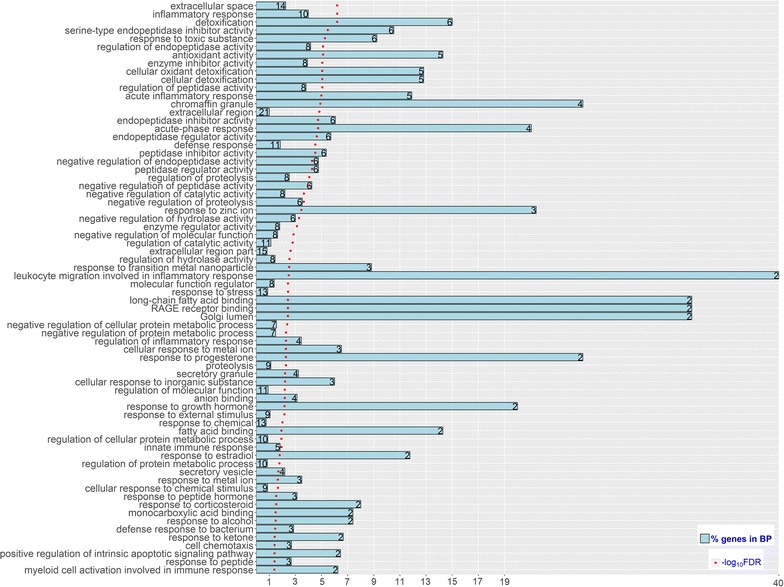
Significantly enriched (FDR < 0.05) biological processes (BP) for the 34 genes detected in the comparison 48 versus −22 h (FDR < 10^−6^; log_2_(fold-change) >2). The significance of enrichment (as −log_10_(FDR)), the % of differentially expressed genes (DEG) over all genes in the BP (as % genes in BP), and the number of DEG in the BP (as the value on *each bar*)

Genomic features can be ranked based on the predictive ability of GFBLUP. Therefore, our GFBLUP can also be used to map gene sets that are associated with complex traits. For instance, a highly up-regulated genomic feature with 34 DEG (FDR < 10^−6^; log_2_(fold-change) > 2) that were detected in the 48 vs. −22 h comparison resulted in an increase of 0.204, 0.020 and 0.041 in prediction accuracy for protein yield among across-breed, and within HOL and JER predictions, respectively (see Additional file 10: Table S15). These 34 DEG, which include <0.01% of the total number of SNPs, explained 1.84 and 4.59% of the genomic variance for protein yield in HOL and JER, respectively. In addition, they explained 0.44 and 0.50% of the genomic variance for mastitis in HOL and JER, respectively, but did not improve genomic predictions for mastitis. Detailed information of GFBLUP analyses for these 34 DEG across three prediction scenarios is in Table 5. The *P* values based on the SNP set test were 0.021 and 0.18 for protein yield and mastitis, respectively, on the HOL training population. The functional enrichment analysis of these 34 DEG revealed that they were significantly (FDR < 0.05) enriched in innate immune response and negative regulation of endopeptidase activity and protein metabolism (Fig. 6).

**Table 5:** GFBLUP analyses of 34 genes detected in the comparison 48 h vs. −22 h (FDR < 10^−6^; log_2_(fold-change) > 2) for mastitis, protein, milk and fat yield

Scenario	Trait	${\text{H}}_{\text{f}}^{2}$ (%)^a^	$r_{\text{GFBLUP}}^{\text{b}}$	*bias* ^c^	Δ*r* ^d^
Within HOL	Mastitis	0.44	0.505	0.865	0.001
	Protein	1.84	0.622	0.783	0.020
	Milk	0.32	0.643	0.863	0.008
	Fat	0.15	0.607	0.809	0.000
Within JER	Mastitis	0.50	0.550	0.918	0.001
	Protein	4.59	0.571	0.797	0.041
	Milk	0.00	0.596	0.789	−0.001
	Fat	0.00	0.434	0.671	0.001
Across-breed	Mastitis	0.46	−0.063	−0.373	−0.005
	Protein	2.24	0.302	1.250	0.204
	Milk	0.25	0.222	0.826	0.062
	Fat	0.09	0.079	0.491	0.009

^a^Proportion of total genomic variance explained by the genomic feature

^b^Prediction accuracy with GFBLUP

^c^Regression of coefficient of de-regressed proofs (DRP) on predicted genomic breeding values (GEBV)

^d^Change in prediction accuracy with GFBLUP relative to GBLUP

## Discussion

In the current study, we demonstrated that a subset of the hepatic transcriptomic regions responsive to IMI was enriched in genomic variants associated with mastitis and milk production traits. When using these regions as genomic features, the genomic prediction accuracy with GFBLUP was improved marginally compared to GBLUP. In theory, both the GFBLUP model and SNP set test can easily be extended to incorporate other types of biological information as genomic features, such as sequence annotation, biological pathways and eQTL.

### Dissection of the genetic architecture and improvement of prediction accuracy for mastitis and milk production traits in dairy cattle

It has been suggested that milk production and disease resistance traits are controlled by several hundred up to several thousand loci in cattle, most of which have a very small effect [4, 49, 50]. Multiple studies, using different strategies, have been conducted to investigate the genetic architecture that underlies such complex phenotypes, and to improve genomic prediction accuracy within and across breeds [6, 17, 49, 51, 52].

#### Genetic architecture and biological interpretation

The approaches that partition genomic variance based on adjacent genomic regions (e.g. 50-SNP genomic segments) or single chromosomes may not provide enough biological insights into the genetic architecture of a trait [6, 51, 53]. Our results provide evidence that results from gene expression experiments can give additional information about the biological and genetic basis of complex traits. In the current study, we used RNA-Seq data from an IMI experiment as an example to study the genetic and biological basis of mastitis and milk production traits. We found that a subset of hepatic transcriptomic regions responsive to IMI is enriched in genomic variants associated with these traits. We also found that down-regulated genes are more often associated with milk and fat yield, which together with the fact that the liver is a crucial organ for host immune responses and metabolism, including lipogenesis, gluconeogenesis, and cholesterol metabolism [54, 55], implies that the immune responses in the liver during mastitis impair milk production. This is in agreement with a recent study that demonstrated that immune relevant pathways (e.g. leukocyte trans endothelial migration and chemokine signalling pathways) are strongly associated with milk and fat yield in HOL [17].

#### Within-breed prediction

In populations with a high degree of linkage disequilibrium (LD), such as highly selected dairy cattle breeds, the genomic relationship based on genome-wide markers provides accurate information about the genomic variation of the traits [56], although it does not use any prior biological information. In addition, the LD structure makes it more difficult to partition genomic variance based on genomic features. Therefore, the increase in prediction accuracy with GFBLUP is small compared to GBLUP, i.e. we observed average increases of 0.018 and 0.022 across four traits within HOL and JER, respectively. This is consistent with a recent study [52] that applied a Bayesian genomic feature model (i.e. BayesRC) to milk production traits. Incorporating 790 candidate genes associated with milk production traits as a genomic feature, they found that the increases in within-breed prediction accuracy with BayesRC were quite small (<0.01) compared to BayesR, which ignores any prior biological information [52].

#### Across-breed prediction

Across-breed genomic prediction accuracies for milk production traits were close to zero, when HOL was used as training population to predict genomic values for JER using the GBLUP approach. This is in agreement with observations in [50, 56]. When validation and training populations are distantly related (i.e. the LD structure becomes weak), genomic feature modelling approaches such as GFBLUP and BayesRC are expected to perform better than models that ignore prior biological information such as GBLUP and BayesR, provided that the genomic feature is enriched in the genomic variants of the traits across breeds [8, 52]. Therefore, shifting the focus from the complete set of genomic markers to those that are more likely to have functional effects might contribute to improve across-breed genomic predictions [7], as observed in our study. However, breed differences in the segregation of quantitative trait loci (QTL), minor allele frequencies and breed-specific SNP effects could add to the complexity in across-breed prediction.

### GFBLUP and alternatives

#### Factors that influence the performance of GFBLUP

The assumption made in the GBLUP model (i.e. the genomic variance is evenly distributed along the whole genome) does not match the real genetic architecture that underlies the traits. It puts equal weights to the elements in the genomic relationship, whereas the GFBLUP allows putting different weights to the individual genomic relationships in the prediction equation according to the estimated genomic parameters [8]. Prediction accuracy of GFBLUP is influenced both by the genomic variance explained by the genomic features and by the number of non-causal SNPs in the feature [8, 9]. The GFBLUP model performs better as the genomic feature contains more causal variants (i.e. explaining more genomic variance) and less non-causal markers [8, 9]. However, if the estimated genomic parameters deviate from the true values, it will lead to reduced prediction accuracy, as shown in the current study (Figs. 3, 4, 5), because too much weight is put on the “wrong” genomic relationships in the prediction equations. Our GFBLUP has two components for genomic effects (i.e. ${\text{f}}$ and $- {\text{f}}$ **)**, but in theory it is possible to include multiple genomic feature effects [57, 58], which might improve genomic predictions more compared to the current GFBLUP. However, when the correlations among multiple genomic relationship matrices are high, the variance components are not reliably estimated and thus there is no improvement in prediction accuracy [8, 57]. Therefore, further work is needed to investigate the performance of the GFBLUP model with multiple genomic features, in particular in livestock populations with large LD structures.

#### Bayesian mixture model and Bayesian GF mixture model

Bayesian mixture models, such as BayesR [50], which ignore prior genomic feature information, are considered to be relevant alternative methods. Both GFBLUP and Bayesian mixture models allow assigning markers to different distributions. GFBLUP assigns a marker set (i.e. genomic feature) to a certain distribution [i.e. ${\text{f}} \sim \,N\left( {0, {\mathbf{G}}_{\text{f}}\upsigma_{\text{f}}^{2} } \right)$ or $- {\text{f}} \sim \,N\left( {0, {\mathbf{G}}_{{ - {\text{f}}}}\upsigma_{{ - {\text{f}}}}^{2} } \right)$] using prior biological knowledge, whereas Bayesian mixture models attempt to assign markers to predefined distributions based on the data themselves. Previous studies demonstrated that an externally informed genomic feature is necessary for a successful partitioning of genomic variance, while the data themselves may not necessarily suggest which marker should have the greatest weight [8, 50]. The external biological information can also be incorporated into Bayesian mixture models, such as BayesRC [52]. All genomic feature models including GFBLUP and BayesRC are computationally intensive, and they do not necessarily perform better than standard models (i.e. GBLUP and BayesR) when genomic features are less enriched in causal variants [8, 59].

### SNP set test

The SNP set test based on single-marker test statistics derived from GWAS is a computationally fast way to evaluate a large number of genomic features [60]. The results of the SNP set test could be used to develop more predictive GFBLUP and similar models. The current SNP set test method assesses the association between a genomic feature and a trait based on the sum of *t* ^2^ of SNPs within the genomic feature. Another commonly used approach for the SNP set test is based on counting associations exceeding a pre-defined significance threshold within the genomic feature [61–63]. One important limitation of this count-based approach is the dichotomization of association signals into significant and non-significant sets, based on a pre-specified significance level, which ignores information regarding the strength of association. Since the genomic variance of mastitis and milk production traits is typically governed by very many markers, each with a small effect [4, 49, 50], the current SNP set test is more likely to match the genetic basis of complex phenotypes, and is more powerful than the count-based approach [9, 45, 46].

#### Appropriate genomic features facilitate improved biological interpretation

In order to test different biological hypotheses, many genomic features can be constructed using different sources of prior information, such as prior QTL regions, chromosomes, sequence, biological pathways, and other types of external evidence. The gain in biological knowledge of complex traits relies highly on the genomic feature classification strategies. Since associated genomic markers are not evenly, or necessarily physically, clustered along the genome [2, 51], partitioning genomic variance based on adjacent genomic regions (e.g. haplotypes and chromosomes) is not an ideal way to facilitate the interpretation of biological mechanisms underlying the traits. Biological interpretation may be better served by the use of pathways and gene ontologies as genomic features; however, the quantity and quality of the genes that are functionally annotated in current pathway databases are limited [15], particularly for livestock and plant genomes. Here, we used information from gene expression data to define genomic features, providing novel insights into the genetic and biological basis of mastitis and milk production traits and improving genomic prediction accuracy with GFBLUP.

Since mastitis can be caused by various pathogens, the current RNA-Seq data that originate only from *E. coli* mastitis may be limited to detect all the genes that are functionally relevant with mastitis. Thus, more RNA-Seq data from infections with other types of pathogens could help the detection of genomic features that are associated with mastitis and milk production. In addition, since gene expression patterns depend highly on time, cell types, and tissues, some trait-associated genes might not show differential expression in certain cell types and tissues at a certain physiological stage. Therefore, incorporating more molecular biological information from more tissues (e.g. mammary gland, blood and adipose tissue) and more physiological stages could be important to define the appropriate genomic features that are highly enriched in causal variants.

## Conclusions

Compared to GBLUP, GFBLUP models increased the accuracy of genomic prediction for mastitis and milk production traits in dairy cattle by incorporating biological information from gene expression data, and thus provide novel biological insights into the genetic basis of such complex traits. Compared to within-breed prediction, the increase in prediction accuracy seems to be more apparent in across-breed prediction. In addition, the SNP set test can be used as a computationally fast way to develop more predictive GFBLUP or similar models. The current genomic feature modelling approaches provide a general framework for incorporating biological knowledge from independent functional genomics studies to study the genetic architecture and to improve genomic prediction for complex traits. Approaches such as GFBLUP and SNP set test will be increasingly useful as the biological knowledge of functional genomic regions keep accumulating for a range of traits and species.

## Additional files

**Additional file 1: Table S1.** The genomic features defined by RNA-Seq analysis. The data provided represent the number of genes in each of the 145 genomic features defined by using six different FDR cut-off values (i.e. ≤5×10^−2^, 10^−2^, 10^−3^, 10^−6^, 10^−8^, and 10^−10^) and four log_2_(fold-change)s (≤−2, ≤−1, ≥1, and ≥2).

**Additional file 2: Table S2.** Gene differential expression analysis of RNA-Seq data. The data provided represent the results of gene differential expression analysis of RNA-Seq data in five different comparisons, i.e. 3 versus −22 h, 6 versus −22 h, 9 versus −22 h, 12 versus −22 h and 48 versus −22 h.

**Additional file 3: Figure S1.** Manhattan plots of single-marker genome-wide association analyses (GWAS) with imputed sequence SNPs. The figure provided represents the *P* values of all imputed sequence SNPs from GWAS for mastitis, protein, milk and fat yield in the HOL training population. Each point represents one SNP.

**Additional file 4: Tables S3, S4, S5 and S6.** GFBLUP and SNP set test analyses in the Holstein (HOL) population. The data provided represent the detailed results of GFBLUP and SNP set test analyses for mastitis (Table S3), protein (Table S4), milk (Table S5) and fat (Table S6) yield in the HOL population.

**Additional file 5: Figure S2.** Relationship between bias of genomic predictions and changes in prediction accuracy with GFBLUP for four traits in the Holstein population. Each point represents one of the 145 genomic features. The y axis is the absolute values of (1-bias (*b*)) for GFBLUP, and the x axis is the changes in prediction accuracy with GFBLUP relative to GBLUP.

**Additional file 6: Tables S7, S8, S9 and S10.** GFBLUP analyses in the Jersey (JER) population. The data provided represent the detailed results of GFBLUP analyses for mastitis (Table S7), protein (Table S8), milk (Table S9) and fat (Table S10) yield in the JER population.

**Additional file 7: Figure S3.** Relationship between bias of genomic predictions and changes in prediction accuracy for four traits in the Jersey (JER) population. Each point represents one of the 145 genomic features. The y axis is the absolute values of (1-bias (*b*)) for GFBLUP, and the x axis is the changes in prediction accuracy with GFBLUP relative to GBLUP.

**Additional file 8: Tables S11, S12, S13 and S14.** Results of GFBLUP analyses across breeds. The data provided represent the detailed results of GFBLUP analyses for mastitis (Table S11), protein (Table S12), milk (Table S13) and fat (Table S14) yield across breeds.

**Additional file 9: Figure S4.** Relationship between bias of genomic predictions and changes in prediction accuracy for four traits in across-breed prediction. Each point represents one of the 145 genomic features. The y axis is the absolute values of (1-bias (*b*)) for GFBLUP, and the x axis is the changes in prediction accuracy with GFBLUP relative to GBLUP.

**Additional file 10: Table S15.** The 34 up-regulated genes associated with protein yield. The data represent the detailed information of the 34 highly up-regulated genes detected in the liver at 48h post intra-mammary infection (IMI).
