# Supplementary material for: Exploring the genetic architecture and improving genomic prediction accuracy for mastitis and milk production traits in dairy cattle by mapping variants to hepatic transcriptomic regions responsive to intra-mammary infection
Source: Genet Sel Evol. 2017 May 12;49:44. doi: 10.1186/s12711-017-0319-0 (PMC5427631; doi:10.1186/s12711-017-0319-0)
Supplement: Supplementary file 2 — Additional file 2: Table S2. Gene differential expression analysis of RNA-Seq data. The data provided represent the results of gene differential expression analysis of RNA-Seq data in five different comparisons, i.e. 3 versus −22 h, 6 versus −22 h, 9 versus −22 h, 12 versus −22 h and 48 versus −22 h. [file 12711_2017_319_MOESM2_ESM.docx]

**Table S2.** The number of genes in each of genomic features identified in intra-mammary infection experiment with LPS based on different FDR cut-offs and log_2_(fold-change)s

|  | Time-point (h) | NG_5E-2_ | NG_1E-2_ | NG_1E-3_ | NG_1E-6_ | NG_1E-8_ | NG_1E-10_ |
| --- | --- | --- | --- | --- | --- | --- | --- |
|  | 3 | 1172 | 826 | 583 | 289 | 192 | 139 |
|  | 6 | 8343 | 6879 | 5422 | 3047 | 2172 | 1638 |
| Total | 9 | 8399 | 6922 | 5559 | 3312 | 2504 | 1923 |
|  | 12 | 7398 | 5864 | 4430 | 2402 | 1768 | 1319 |
|  | 48 | 485 | 240 | 128 | 48 | 32 | 25 |
|  | 3 | 848 | 641 | 482 | 260 | 176 | 128 |
|  | 6 | 4149 | 3518 | 2909 | 1790 | 1335 | 1039 |
| Up-1 | 9 | 4112 | 3440 | 2770 | 1602 | 1159 | 888 |
|  | 12 | 3647 | 2863 | 2126 | 1009 | 689 | 478 |
|  | 48 | 325 | 179 | 107 | 47 | 32 | 25 |
|  | 3 | 290 | 259 | 230 | 169 | 137 | 113 |
|  | 6 | 1205 | 1127 | 1056 | 863 | 753 | 662 |
| Up-2 | 9 | 902 | 841 | 773 | 631 | 554 | 502 |
|  | 12 | 663 | 587 | 530 | 375 | 331 | 264 |
|  | 48 | 86 | 66 | 48 | 34 | 29 | 24 |
|  | 3 | 324 | 185 | 101 | 29 | 16 | 11 |
|  | 6 | 4194 | 3361 | 2513 | 1257 | 837 | 599 |
| Down-1 | 9 | 4287 | 3482 | 2789 | 1710 | 1345 | 1035 |
|  | 12 | 3751 | 3001 | 2304 | 1393 | 1079 | 841 |
|  | 48 | 160 | 61 | 21 | 1 | 0 | 0 |
|  | 3 | 56 | 39 | 26 | 13 | 10 | 9 |
|  | 6 | 836 | 758 | 686 | 534 | 449 | 382 |
| Down-2 | 9 | 1288 | 1157 | 1060 | 888 | 800 | 707 |
|  | 12 | 1067 | 978 | 896 | 751 | 679 | 603 |
|  | 48 | 33 | 16 | 9 | 0 | 0 | 0 |

NG_5E-2_, NG_1E-2_, NG_1E-3_, NG_1E-6_, NG_1E-8_ and NG_1E-10_ represent the number of genes detected based on FDR < 5×10^-2^, 10^-2^, 10^-3^, 10^-6^, 10^-8^ and 10^-10^ respectively. Up-1 and Up-2 represent up-regulation with log2 (fold-change) > 1 and 2 respectively, while Down-1 and Down-2 represent down-regulation with log2(fold-change) < -1 and -2 respectively.
