# Supplementary figures and images for: Exploring the genetic architecture and improving genomic prediction accuracy for mastitis and milk production traits in dairy cattle by mapping variants to hepatic transcriptomic regions responsive to intra-mammary infection
Source: Genet Sel Evol. 2017 May 12;49:44. doi: 10.1186/s12711-017-0319-0 (PMC5427631; doi:10.1186/s12711-017-0319-0)

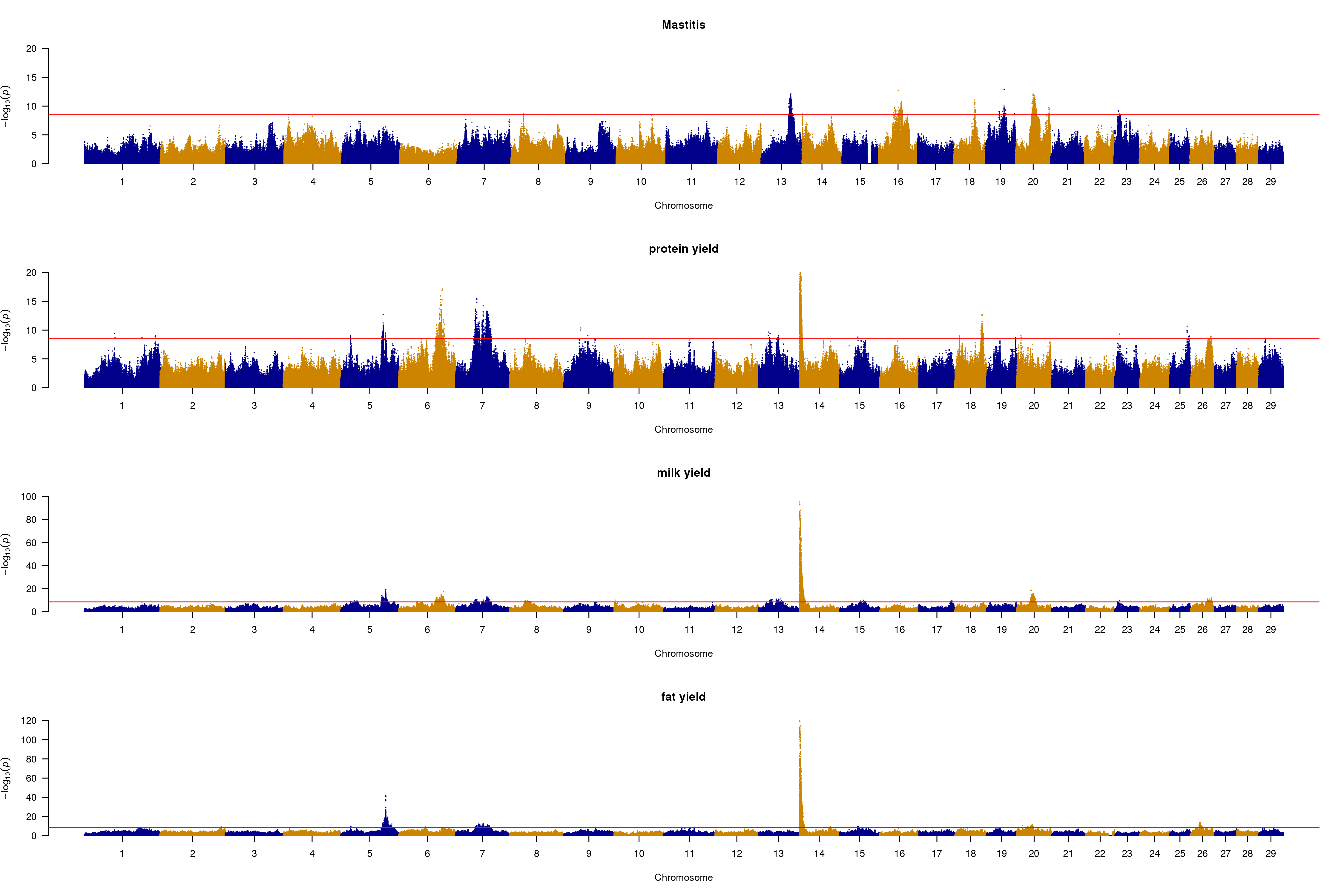

Supplement: Supplementary file 3 — Additional file 3: Figure S1. Manhattan plots of single-marker genome-wide association analyses (GWAS) with imputed sequence SNPs. The figure provided represents the P values of all imputed sequence SNPs from GWAS for mastitis, protein, milk and fat yield in the HOL training population. Each point represents one SNP. [file 12711_2017_319_MOESM3_ESM.tiff]

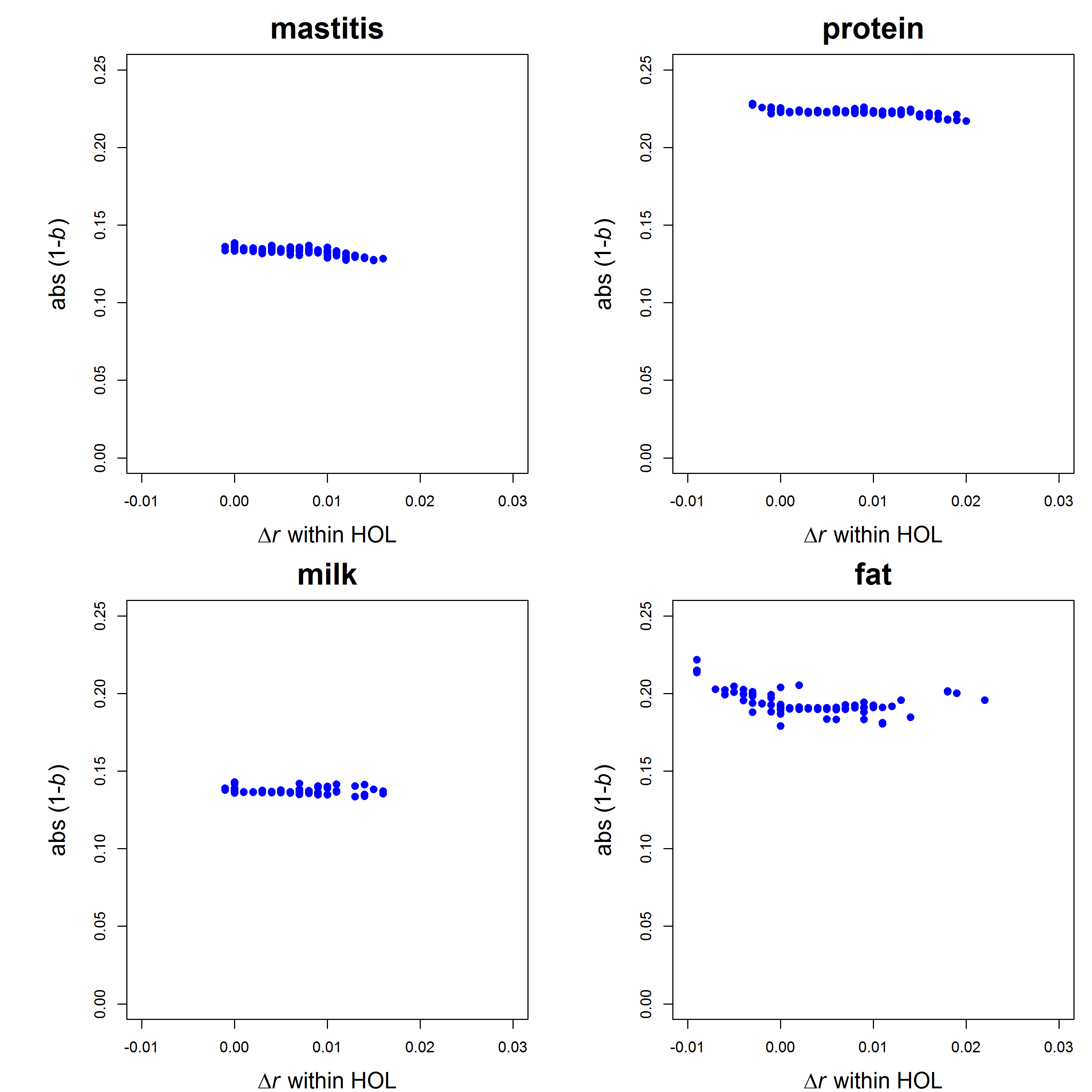

Supplement: Supplementary file 5 — Additional file 5: Figure S2. Relationship between bias of genomic predictions and changes in prediction accuracy with GFBLUP for four traits in the Holstein population. Each point represents one of the 145 genomic features. The y axis is the absolute values of (1-bias (b)) for GFBLUP, and the x axis is the changes in prediction accuracy with GFBLUP relative to GBLUP. [file 12711_2017_319_MOESM5_ESM.tiff]

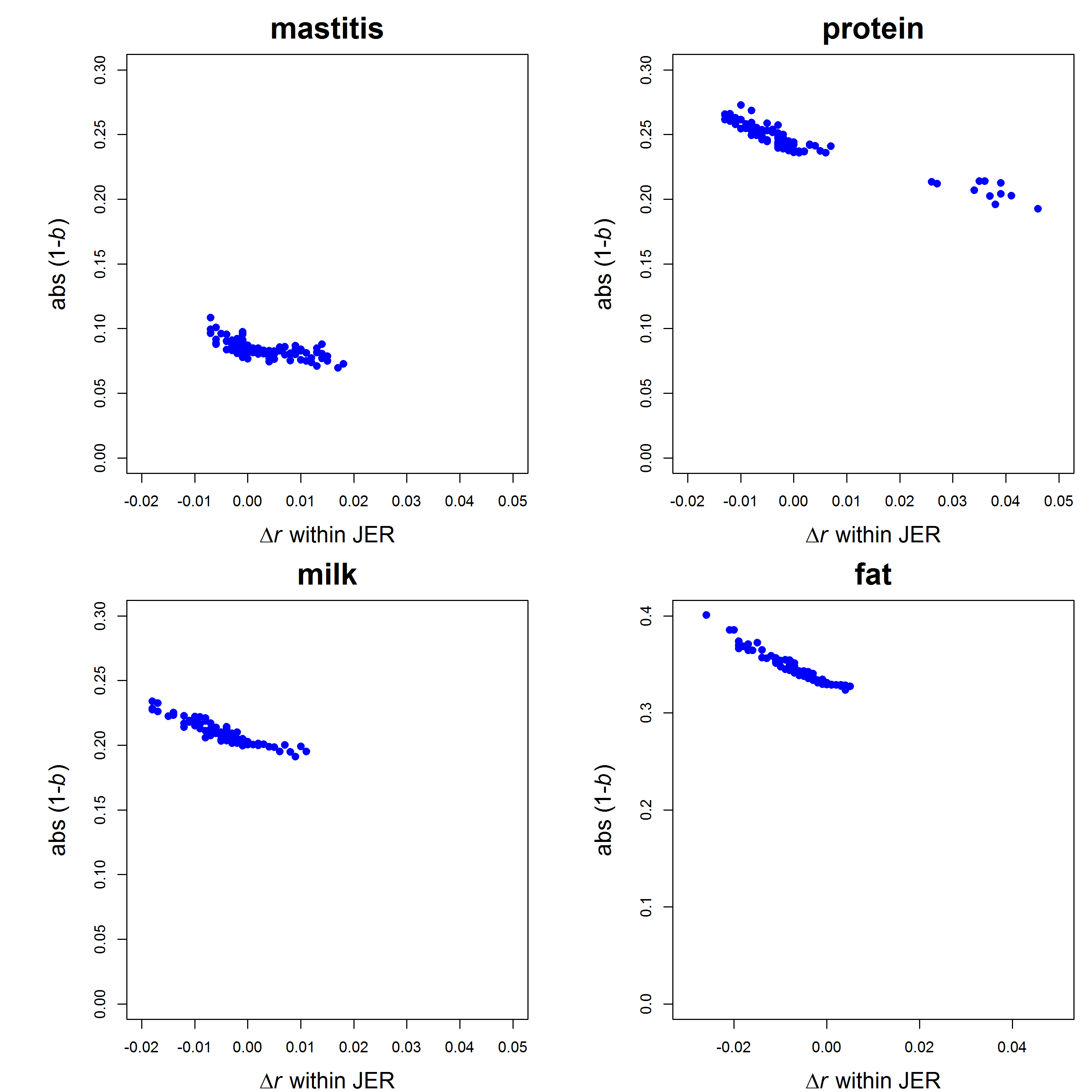

Supplement: Supplementary file 7 — Additional file 7: Figure S3. Relationship between bias of genomic predictions and changes in prediction accuracy for four traits in the Jersey (JER) population. Each point represents one of the 145 genomic features. The y axis is the absolute values of (1-bias (b)) for GFBLUP, and the x axis is the changes in prediction accuracy with GFBLUP relative to GBLUP. [file 12711_2017_319_MOESM7_ESM.tiff]

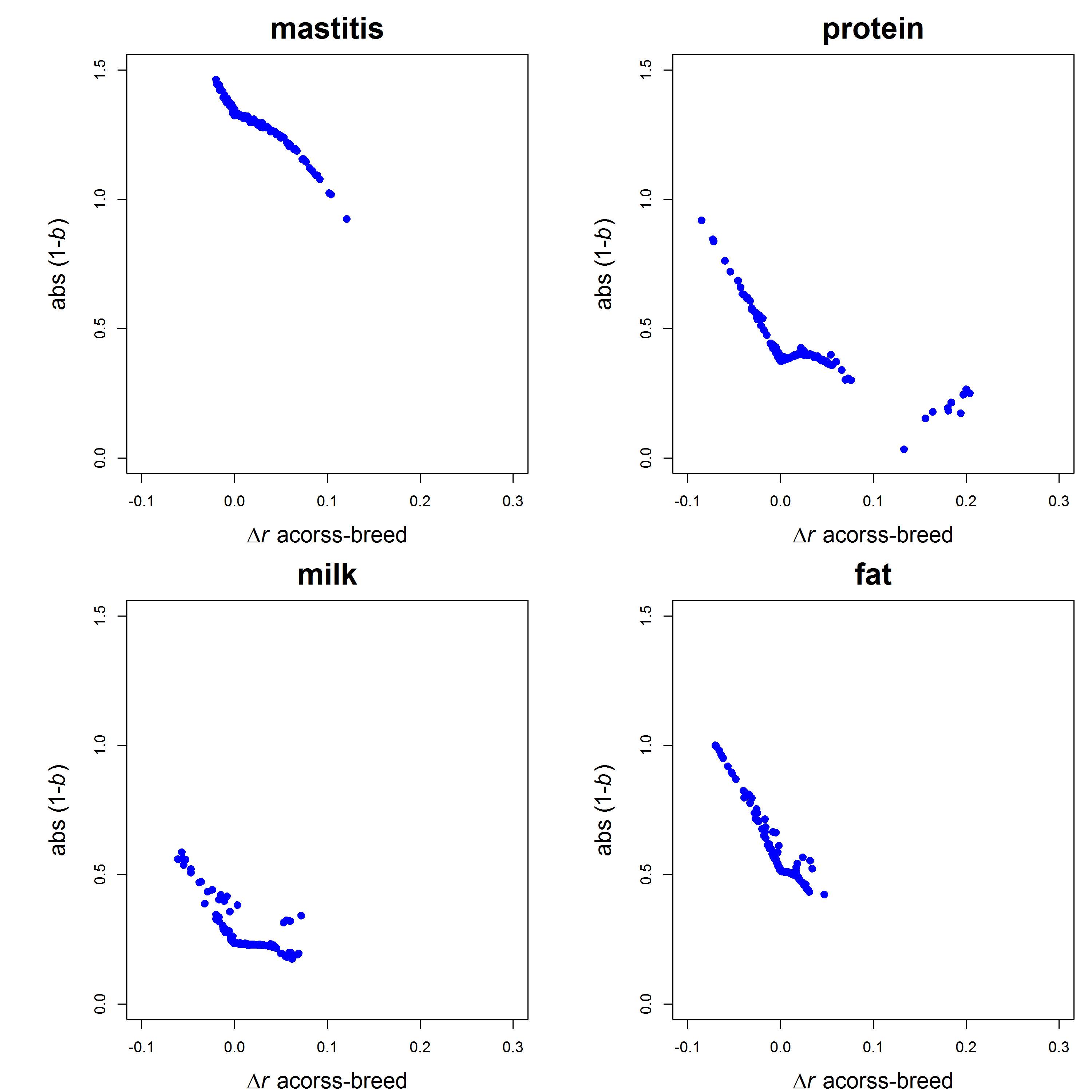

Supplement: Supplementary file 9 — Additional file 9: Figure S4. Relationship between bias of genomic predictions and changes in prediction accuracy for four traits in across-breed prediction. Each point represents one of the 145 genomic features. The y axis is the absolute values of (1-bias (b)) for GFBLUP, and the x axis is the changes in prediction accuracy with GFBLUP relative to GBLUP. [file 12711_2017_319_MOESM9_ESM.tiff]
